# Mechanistic insights into the renoprotective role of curcumin in cisplatin-induced acute kidney injury: network pharmacology analysis and experimental validation

**DOI:** 10.1080/21655979.2021.2005916

**Published:** 2021-11-27

**Authors:** Hui Zhang, Qing-Qing Dong, Hua-Pan Shu, Yu-Chi Tu, Qian-Qian Liao, Li-Jun Yao

**Affiliations:** Department of Nephrology, Union Hospital, Tongji Medical College, Huazhong University of Science and Technology, Wuhan, China

**Keywords:** Cisplatin, acute kidney injury, curcumin, network pharmacology, molecular docking, Akt, apoptosis

## Abstract

Cisplatin-induced acute kidney injury (CP-AKI) is a severe complication in patients receiving CP chemotherapy. However, effective therapies for CP-AKI are currently lacking. Curcumin (CUR), a natural polyphenol, is extracted from the rhizome of turmeric and has been reported to have nephroprotective activity. However, the role of CUR in CP-AKI remains unclear. This study aimed to explore the mechanism of CUR in CP-AKI by combining a network pharmacology approach with experimental validations. The analysis revealed 176 potential targets of CUR based on the HERB database and 1,286 related targets of CP-AKI from the GeneCards, DrugBank, and OMIM databases. Further, 106 common targets of CUR against CP-AKI were obtained, and these common targets constructed a protein-protein interaction (PPI) network. In addition, the core targets were screened from the PPI network using Cytoscape. Molecular docking revealed that CUR displayed the best binding to AKT1. Gene Ontology (GO) analysis indicated that the primary biological processes of CUR against CP-AKI included cellular response to chemical stress and apoptotic regulation. Kyoto Encyclopedia of Genes and Genomes (KEGG) pathway enrichment analysis suggested that the PI3K-Akt signaling pathway was most significantly enriched in CUR against CP-AKI. Western blotting and flow cytometry showed that CUR inhibited apoptosis induced by CP by activating the Akt signaling pathway in human kidney tubular epithelial cells (HK-2). Altogether, our findings demonstrated that CUR alleviated apoptosis by activating the Akt signaling pathway in CP-AKI *in vitro*. These data provide a scientific basis for future investigations into the clinical application of CUR against CP-AKI.

## 1. Introduction

Acute kidney injury (AKI), a global health problem with increasing morbidity and mortality year by year, is characterized by a rapid accumulation of metabolic waste products, such as urea and a decline in renal function [1]. Clinically, the three most common causes of AKI include ischemia-reperfusion injury, sepsis, and nephrotoxins. Nephrotoxic medications contribute to approximately 20% of AKI patients [2]. Cisplatin (CP), a first-line agent for the treatment of several cancers, is eliminated from the kidney and accumulates in renal tubular cells, resulting in tubular cell injury and death. As a result, nephrotoxicity often limits the dose of CP [3]. Approximately 30% of patients undergoing CP treatment develop renal dysfunction, with AKI as the most common manifestation [4]. Further, the pathophysiology of CP-AKI mainly involves autophagy, oxidative stress, inflammation, and apoptosis [5]. The current therapies for CP-AKI, such as hydration and forced diuresis, are interventions for symptomatic but not disease courses based on pathological mechanisms. Moreover, these therapies are associated with a series of side effects [6]. Therefore, there is an urgent need to discover safer and more effective medicines.

Curcumin (CUR) is a phenolic compound isolated from *Curcuma longa* and is known for its multiple pharmacological activities, including anti-inflammatory, antioxidant, and apoptotic regulation properties [7–9]. Extensive studies have demonstrated that CUR exerts renoprotective effects on multiple kidney diseases, such as chronic kidney disease, ischemia-reperfusion AKI, and nonalcoholic steatohepatitis kidney disease [10–13]. Furthermore, CUR has been reported to significantly improve CP-induced reduction of kidney function by suppressing oxidative stress injury, inflammatory and apoptotic responses in rats [14]. Although a few studies have reported the protective effect of CUR on CP-AKI, the molecular mechanisms involved are still largely unknown.

Network pharmacology is an emerging and promising approach. It can integrate bioinformatics with pharmacology to provide persuading evidence for drugs on disease through visualization [15–18]. This study aimed to explore the potential targets and pathways of CUR against CP-AKI through a network pharmacological approach. On the other hand, CP-treated HK-2 cells were used as an in vitro AKI model to validate the function and mechanism of CUR in regulating apoptosis. Our results show that CUR alleviates CP-mediated apoptosis at least partially through activation of Akt signaling.

## 2. Material and methods

**Figure 1.**
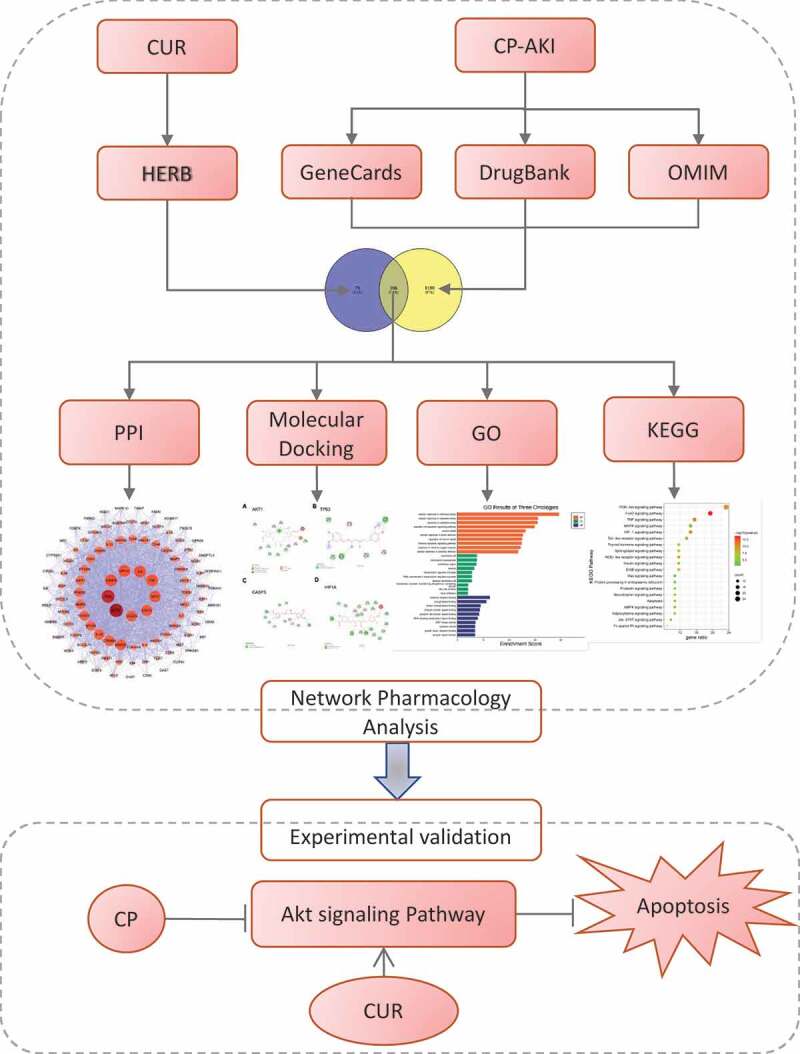
The flow chart of this study

The protocol of this study is shown in Figure 1.

### 2.1. Screening for the potential targets of CUR

The target proteins of CUR were collected from the HERB Database (http://herb.ac.cn/, accessed on 24 August 2021) [19]. This database integrates multiple Traditional Chinese Medicine (TCM) databases and supports TCM data based on high-throughput experiments and reference-guidance, guaranteeing the credibility of targets related to CUR. The 2D structure of CUR was acquired from the PubChem database (https://pubchem.ncbi.nlm.nih.gov/, accessed on 24 August 2021) [20].

### 2.2. Target identification for CP-AKI

Disease-related targets were obtained from the GeneCards database (https:// www.genecards.org, accessed on 24 August 2021) [21], the Drug Bank database (https://www.drugbank.ca/, accessed on 24 August 2021) [22], and the Online Mendelian Inheritance in Man (OMIM) database (https://omim.org/, accessed on 24 August 2021) [23] using the keywords: ‘cisplatin-induced acute kidney injury,’ ‘cisplatin-induced acute renal injury,’ ‘cisplatin-induced acute kidney failure,’ and ‘cisplatin-induced acute renal failure.’ After merging the results of these databases and removing duplicate targets, the related targets of CP-AKI were identified.

### 2.3. Screening common targets of CUR and CP-AKI

The common targets between CUR and CP-AKI were intersected using the VENNY 2.1 online platform (https://bioinfogp.cnb.csic.es/tools/venny/, accessed on 24 August 2021) [24].

### 2.4. Protein-protein interaction (PPI) network construction

The PPI network of common targets was constructed using the STRING database (https://string-db.org/, accessed on 24 August 2021) [25]. The organism was set to *Homo sapiens*; confidence scores were larger than 0.4, and the other variables remained as the default. Thereafter, the PPI network was exported in TSV format and analyzed using Cytoscape 3.7.2 (https://cytoscape.org/) [26]. PPI networks were formed with nodes (indicating a target protein) and edges (indicating PPI). The degree indicates the number of nodes directly connected to a node. Core targets were identified using the Cytoscape plugin (Network Analysis). Herein, the top 10 proteins by degree value ranking were selected as core targets.

### 2.5 Molecular docking

The 3D structures of the top four proteins by the degree value of core targets were achieved from the Protein Data Bank database (https://www.rcsb.org/, accessed on 25 August 2021) [27], AKT1 (PDB ID: 3096, TP53 (PDB ID: 2k8f), CASP3 (PDB ID: 3DEH), and HIF1A (PDB ID:5L9V). Water molecules and unwanted ligands from the selected target proteins were removed using PyMOL™ 2.5.2 [28]. The receptor proteins were modified using AutoDock tools [29]. Docking of the receptor and small molecular ligand was performed using AutoDock Vina 1.1.2 [29]. The 2D complex and interactions of the docked ligand and receptor were analyzed using Discovery Studio Visualizer Version Client [30]. Affinity scores were used to evaluate the binding potential between the top four core targets and CUR.

### 2.6. Gene function and pathway enrichment analysis

Gene Ontology (GO) functional enrichment analysis and Kyoto Encyclopedia of Genes and Genomes (KEGG) pathway enrichment analysis for common targets were performed using DAVID 6.8 (https://david.ncifcrf.gov/, accessed on 25 August 2021) [31]. The enriched terms with *p* < 0.05 and FDR < 0.05 were considered significant. The top 20 enriched terms were visualized using an online tool (www.bioinformatics.com.cn, accessed on 25 August 2021) [32]. A target-pathway network of CUR against CP-AKI was constructed using Cytoscape software.

### 2.7. Cell culture and treatment

Human kidney tubular epithelial cells (HK-2) with normal biochemical features and proximal tubular cell morphology were purchased from the American Type Culture Collection (ATCC; Manassas, VAm USA) and were cultured in a DMEM medium at a 37°C incubator in 5% CO2. The DMEM medium was supplemented with 10% FBS (Sciencell, San Diego, CA, USA), streptomycin (100 mg/mL), and penicillin (100 IU/mL) (Thermo Fisher Scientific, Waltham, MA, USA).

The cells were starved of serum for 12 h before treatment during the experiments. CP was purchased from Sigma (St. Louis, MO, USA) and was administered to HK-2 cells at a concentration of 20 µM for 24 h to establish the CP-AKI cell model [33–35]. CUR was purchased from Dalian Meilun Biotechnology (C21H20O6; purity >97%). DMSO was used as a solvent control (CON) for CUR; the same volume of DMSO was treated to cells as a control. When an appropriate confluency was reached, HK-2 cells were treated with four concentrations of CUR (0, 5, 10, and 20 µM) for 24 h. A 10 µM concentration of CUR was selected as the final stimulation concentration for subsequent experiments. The activity of Akt was inhibited using a specific inhibitor (VIII; Topsicence, Shanghai, China). DMSO was used as a solvent control for Akt inhibitor VIII treatment. Cells treated with 10 µM VIII for 24 h were used as specified for each experiment.

### 2.8. Cell viability assay

According to the manufacturer’s instructions, viability was assessed using CCK-8 kits (Beyotime, Shanghai, China). Cells were cultured in 96-well microplates at a density of 5 × 10^3^/well in 100 μL of medium and treated with four concentrations (0, 5, 10, and 20 µM) of CUR for 24 h. Subsequently, 10 μL of CCK-8 reagent was added to each well and incubated for 2 h. The absorbance at 450 nm was measured, indicating cell viability. All experiments were performed in triplicate. All assays were repeated seven times. Wells without cells served as blanks.

### 2.9. Apoptosis and cell cycle assays

An Annexin V-FITC/Propidium Iodide apoptosis kit (Keygentec, China) was used to evaluate cell apoptosis. Treated HK-2 cells were cultured to a density of 80–90% in 6-well plates and collected following trypsin digestion. Cultured cells were washed with PBS and then were collected for detection. Apoptosis was detected by resuspending the cells in 500 μL of binding buffer. A mixture of 5 μL PI and 5 μL of annexin V-FITC was added to the binding buffer and incubated for 30 min in the dark at room temperature. Subsequently, cell apoptosis was examined using flow cytometry. Apoptosis in the early stage was indicated by PI-negative/Annexin V-positive staining, while more advanced apoptosis was indicated by PI-positive/annexin V-positive staining. The cell experiments were repeated in triplicate. The apoptotic data of flow cytometry was analyzed using FlowJo 10 software.

### 2.10. Western blot (WB) analysis

HK-2 cells were lysed with the lysis buffer of the radioimmunoprecipitation assay (RIPA) (Beyotime, Shanghai, China) in accordance with standard procedures. The BCA protein assay kit (Thermo Scientific, Rockford, IL, USA) was used to calculate the protein concentration. The proteins were separated by SDS-polyacrylamide gel electrophoresis (8–10%) and transferred to polyvinylidene fluoride membranes (Millipore Corp, Bedford, MA, USA). The membranes were blocked with 5% skimmed milk (Beyotime, Shanghai, China) for 2 h and then incubated with primary antibodies against β-actin (Proteintech, Rosemont, IL, USA), Bcl2, Bax, cleaved caspase-3, p-Akt (Ser-473) and Akt (Cell Signaling Technology, Danvers, MA, USA).

### 2.11. Statistical analysis

Statistical analysis was performed using SPSS 20.0 software (IBM Corp., Armonk, NY, USA). All data were expressed as mean ± SD. All experiments were repeated in triplicate. The normality of the data was examined using Shapiro–Wilk test. For data variables with normal distribution and independent samples, Student’s unpaired t-test was used to compare the two groups [36], while one-way analysis of variance (ANOVA) with Dunnett’s post-hoc test was applied to compare more than two groups [37,38]. Statistical significance was set at *p *< 0.05.

## 3. Results

The protective effects of CUR against CP-AKI and the possible mechanisms of action have not been fully elucidated. Here, we used a network pharmacology approach to predict the potential targets and candidate pathways of CUR involvement in CP-AKI. CP-induced HK-2 cells were used as a cellular model to validate the role and mechanism of CUR against CP-AKI.

### 3.1. The pharmacological properties of CUR and PPI network analysis of shared targets of CUR and CP-AKI

**Figure 2.**
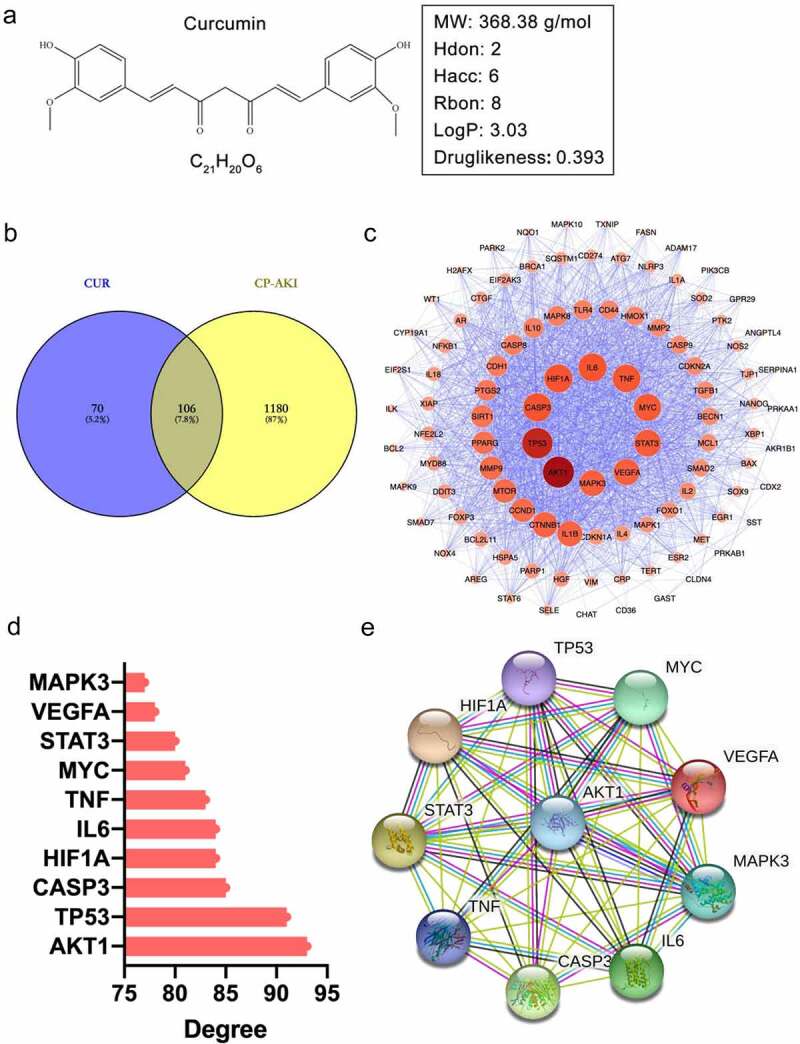
The pharmacological properties of CUR and PPI network analysis of shared targets from CUR and CP-AKI. (a) 2D structure of curcumin (CUR) and the corresponding pharmacological properties. (b) The Venn diagram acquired an intersection between CUR and CP-AKI. The purple area indicates targets for CUR, the yellow zone indicates targets for CP-AKI, and the overlap zone indicates the common targets. (c) PPI network of CUR against CP-AKI. Nodes represent targets protein; edges indicate interaction of targets. The larger the node and the deeper the color, the higher the degree. (d) The top 10 core targets ranked by degree-value. (e) PPI network analysis of core targets

We obtained the 2D structure of CUR from the PubChem database (Figure 2(a)) and assessed the pharmacokinetic properties of CUR using the online tool, SwissADME. Generally, a compound that satisfied the requirements of Lipinski’s rule of five is more likely to become a drug [39]. SwissADME prediction showed that CUR complies with Lipinski’s rule of five: molecular weight (MW), 368.38 g/mol; hydrogen bond donors (Hdon), 2; hydrogen bond acceptors (Hacc), 6; rotatable bonds (Rbon), 8; lipid-water partition coefficient (log P), 3.03; and the Drug-likeness weight of CUR, 0.393, as obtained from the Encyclopedia of Traditional Chinese Medicine (ETCM, http://www.tcmip.cn/ ETCM/). In general, a drug-likeness weight ≥ 0.18 indicates good drug-likeness. Collectively, these results suggest that CUR has good pharmacokinetic properties.

By searching the high-throughput experiment and reference-guided database, HERB, 176 targets of CUR were screened. A total of 1,286 potential targets of CP-AKI were obtained from the GeneCards, DrugBank, and OMIM databases. By intersecting the targets of CUR with CP-AKI, 106 shared targets were identified (Figure 2(b)). To investigate the interactions of the 106 common targets, we constructed a PPI network using the STRING database and Cystoscape software. In the PPI network, 102 nodes and 2,056 edges with an average degree value of 40.3 were obtained. The darker the color and the larger the node, the higher the degree value. Among the nodes, the top 10 targets by degree value were recognized as core targets, including AKT1 (93), TP53 (91), CASP3 (85), HIF1A (84), IL6 (84), TNF (83), MYC (81), STAT3 (80), VEGFA (78), and MAPK3 (77) (Figures 2**(c), 2**(d)). These core targets formed another PPI network consisting of 10 nodes and 36 edges with an average degree of 8 (Figure 2(e)). These results indicate that the core targets could significantly interact with other targets in the PPI network, revealing that these targets may play a crucial role in the pathological process of CP-AKI.

### 3.2. Molecular docking analysis

**Figure 3.**
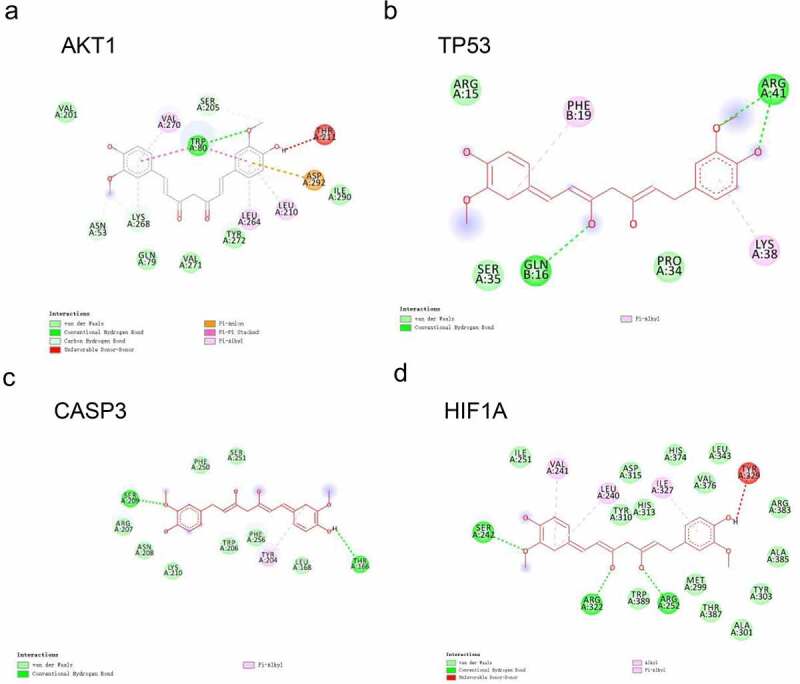
Docking analysis between CUR and the top four core targets: AKT1, TP53, CASP3, and HIF1A (ranked by degree-value) (a–d). The deep green dashed lines indicate hydrogen bonds, light pink dashed lines suggest pi-alkyl, deep pink dashed lines represent pi-pi stack, and brown dashed lines indicate pi-Anion interactions. The light green nodes represented the Vander Waals interactions. The dark red nodes indicate unfavorable donor-donor

To verify the binding activity of CUR to the targets, we selected the top four core targets by degree value for molecular docking, including AKT1, TP53, CASP3, and HIF1A. We summarized the affinities of CUR with the interactive residues of the selected targets and predicted the number of hydrogen bonds formed between interactive residues. The docking results are shown in Table 1 and Figure 3(a-d).

**Table 1.** Molecular docking of the top four core targets by degree with CUR

Integration	Targets	PDB ID	Affinity (kcal/mol)	H bonds	H bond interacting residues
CUR	AKT1	3096	−9.5	1	TRP80
	TP53	2k8f	−6.6	3	ARG4(2), GLN16
	CASP3	3DEH	−6.7	2	SER209, THR166
	HIF1A	5L9V	−8.9	3	SER242, ARG322, ARG252

*Notes*: TRP, Tryptophan; ARG, Arginine; GLN, Glutamine; SER, Serine; THR, Threonine.

#### 3.2.1. Docking of CUR on AKT1

The binding energy of CUR on AKT1 was – 9.5 kcal/mol. As shown in Figure 3(a), the TRP80 residue provided one hydrogen bond and two pi-pi stacked interactions with CUR. In addition, the VAL201, GLN79, VAL271, TYR272, and ILE290 residues constituted five van der Waals interactions. The ASP292 residue was found to form a pi-anion, and the LYS268, VAL270, LEU264, and LEU210 residues were formed by pi-alkyl interactions.

#### 3.2.2. Docking of CUR on TP53

The binding energy of CUR on TP53 was – 6.6 kcal/mol. As shown in Figure 3(b), three hydrogen bonds were provided by ARG41 (two H bonds) and GLN16 (one H bond). In addition, the ARG15, SER35, and PRO34 residues constituted three van der Waals interactions. The PHE19 and LYS38 residues formed two pi-alkyl bonds.

#### 3.2.3. Docking of CUR on CASP3

The binding energy of CUR on CASP3 was – 6.7 kcal/mol. As shown in Figure 3(c), two hydrogen bonds were provided by the SER209 and THR166 residues in the interaction with CUR. In addition, the PHE250, SER251, ARG207, ASN208, LYS210, TRP206, PHE256, and LEU168 residues constituted eight van der Waals interactions. The TYR204 residue formed one pi-alkyl bond.

#### 3.2.4. Docking of CUR on HIF1A

The binding energy of CUR on HIF1A was – 8.9 kcal/mol. As shown in Figure 3(d), three hydrogen bonds were provided by the SER242, ARG322, and ARG252 residues in the interaction with CUR. Further, the ILE251, ASP315, TYR310, HIS313, HIS374, LEU343, VAL376, ARG383, ALA385, TYR303, MET299, THR387, ALA301, and TRP389 residues constituted 14 van der Waals interactions. The VAL241, LEU240, and ILE327 residues formed three pi-alkyl bonds.

With respect to the interaction between the molecule and target, the binding energy indicates the affinities. The more negative the binding energy, the stronger the interaction [40]. A binding energy lower than – 5.0 kcal/mol indicates good binding activity, while a binding energy lower than – 7.0 kcal/mol suggests a stronger binding force and more significant interaction. Our results revealed that the binding energy of CUR to the four selected core targets was less than – 5.0 kcal/mol, which indicates a good interaction between CUR and the top four core targets (AKT1, TP53, CASP3, HIF1A). Among the top four core targets, AKT1 exhibited the lowest binding energy of – 9.5 kcal/mol, and thus has the best binding affinity with CUR. These results show that CUR significantly binds to the four core targets.

### 3.3. GO and KEGG enrichment analysis

**Figure 4.**
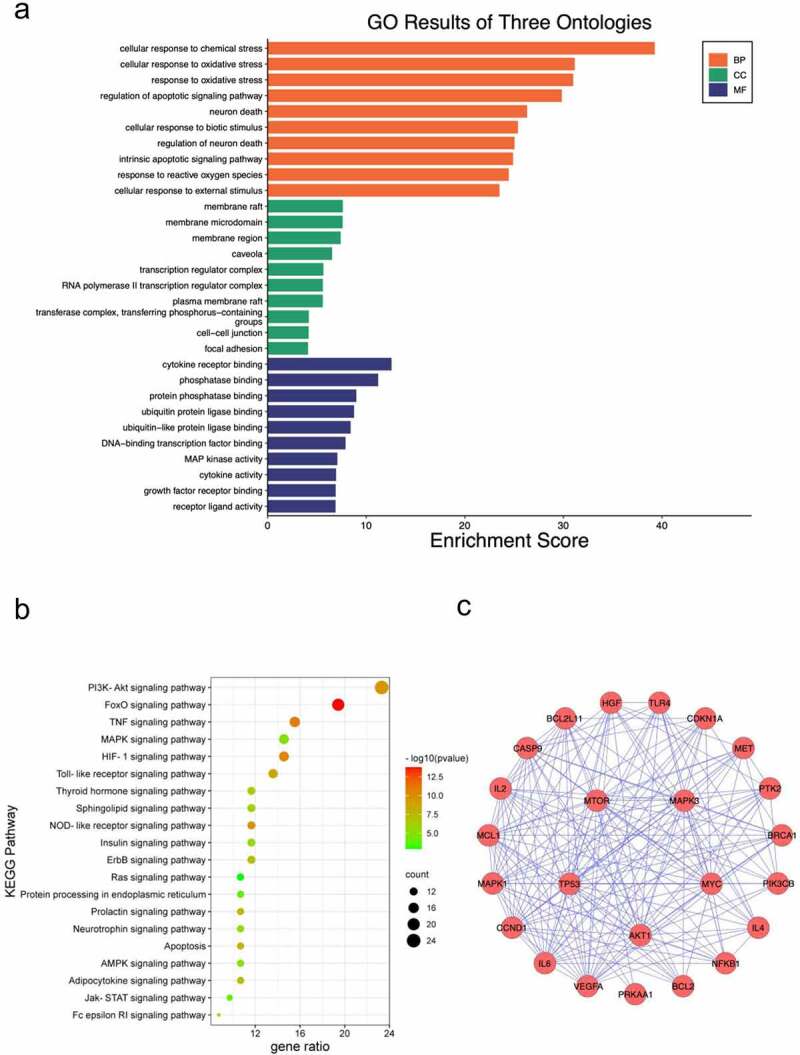
GO enrichment analysis and KEGG pathway enrichment analysis of CUR against CP-AKI. (a) The biological process (BP), cell composition (CC), and molecular function (MF) of GO enrichment analysis were represented by brown columns, green columns, and blue columns, respectively. The X-axis represents the enrichment score, and the Y-axis indicates the category of GO. (b) The top 20 KEGG pathways are presented in the bubble chart. The X-axis shows the gene ratio, the color represents the p-value, and bubble size represents the count of targets enriched in terms. (c) The PPI network of targets involved in the PI3K-Akt signaling pathway

To explore the underlying mechanism of CUR against CP-AKI, GO and KEGG enrichment analysis were carried out using the DAVID database. The main GO biological processes (BP) included cellular response to chemical stress, cellular response to oxidative stress, regulation of apoptotic signaling pathway, among others. For cell composition (CC), the membrane raft, membrane microdomain, and membrane region were the main classifications of these targets. Molecular function (MF) category included cytokine receptor binding, phosphatase binding, and ubiquitin-protein ligase binding (Figure 4(a)). The primary enriched KEGG pathways were the PI3K-Akt signaling pathway, FOXO signaling pathway, TNF signaling pathway, MAPK signaling pathway, HIF-1 signaling pathway, among others (Figure 4(b)). Detailed data of KEGG enrichment are provided in Table 2. The enriched PI3K-Akt signaling pathway included 24 targets of CUR against CP-AKI, and these targets formed a complicated PPI network that consisted of 24 nodes and 163 edges, with an average degree value of 13.6 (Figure 4(c)).

**Table 2.** Top 20 terms of KEGG pathways enriched in CUR against CP-AKI

Term	Pathway	Gene ratio (%)	*p*-value	Count	Symbols
hsa04151	PI3K-Akt signaling pathway	23.30	2.32E-10	24	CDKN1A, PRKAA1, HGF, BRCA1, PIK3CB, IL2, MTR, PTK2, NFKB1, VEGFA, CASP9, IL4, IL6, BCL2L11, CCND1, MYC, BCL2, AKT1, MAPK1, MET, TP53, TLR4, MCL1, MAPK3
hsa04068	FOXO signaling pathway	19.42	1.69E-14	20	IL10, SMAD2, CDKN1A, PRKAA1, TGFB1, STAT3, PIK3CB, SD2, SIRT1, PRKAB1, FX1, MAPK10, MAPK9, IL6, MAPK8, BCL2L11, CCND1, AKT1, MAPK1, MAPK3
hsa04668	TNF signaling pathway	15.53	1.50E-11	16	PIK3CB, PTGS2, SELE, TNF, MMP9, NFKB1, MAPK10, MAPK9, IL6, MAPK8, CASP8, IL1B, CASP3, AKT1, MAPK1, MAPK3
hsa04066	HIF-1 signaling pathway	14.56	4.37E-11	15	CDKN1A, NS2, STAT3, PIK3CB, HIF1A, MTR, NFKB1, VEGFA, IL6, BCL2, AKT1, HMX1, MAPK1, TLR4, MAPK3
hsa04010	MAPK signaling pathway	14.56	1.05E-05	15	TGFB1, TNF, NFKB1, MAPK10, IL1A, MAPK9, MAPK8, MYC, IL1B, CASP3, DDIT3, AKT1, MAPK1, TP53, MAPK3
hsa04620	Toll-like receptor signaling pathway	13.59	2.08E-09	14	PIK3CB, TNF, NFKB1, MAPK10, MAPK9, IL6, MAPK8, CASP8, IL1B, AKT1, MAPK1, TLR4, MYD88, MAPK3
hsa04621	NOD-like receptor signaling pathway	11.65	2.17E-10	12	MAPK10, MAPK9, IL6, MAPK8, CASP8, IL1B, IL18, NLRP3, MAPK1, TNF, NFKB1, MAPK3
hsa04012	ErbB signaling pathway	11.65	2.85E-08	12	MAPK10, MAPK9, CDKN1A, MAPK8, MYC, MAPK1, AKT1, PIK3CB, AREG, PTK2, MTR, MAPK3
hsa04919	Thyroid hormone signaling pathway	11.65	5.24E-07	12	CASP9, CCND1, MYC, MAPK1, CTNNB1, AKT1, PIK3CB, HIF1A, TP53, FX1, MTR, MAPK3
hsa04071	Sphingolipid signaling pathway	11.65	8.06E-07	12	MAPK10, MAPK9, MAPK8, BCL2, BAX, MAPK1, AKT1, PIK3CB, TNF, TP53, NFKB1, MAPK3
hsa04910	Insulin signaling pathway	11.65	3.24E-06	12	MAPK10, MAPK9, PRKAA1, MAPK8, FASN, MAPK1, AKT1, PIK3CB, PRKAB1, FX1, MTR, MAPK3
hsa04210	Apoptosis	10.68	1.14E-08	11	CASP9, CASP8, CASP3, BCL2, BAX, XIAP, AKT1, PIK3CB, TNF, TP53, NFKB1
hsa04920	Adipocytokine signaling pathway	10.68	3.83E-08	11	MAPK10, MAPK9, PRKAA1, MAPK8, STAT3, AKT1, CD36, TNF, PRKAB1, NFKB1, MTR
hsa04917	Prolactin signaling pathway	10.68	4.41E-08	11	MAPK10, MAPK9, MAPK8, CCND1, STAT3, MAPK1, AKT1, PIK3CB, ESR2, NFKB1, MAPK3
hsa04722	Neurotrophic signaling pathway	10.68	6.27E-06	11	MAPK10, MAPK9, MAPK8, BCL2, BAX, MAPK1, AKT1, PIK3CB, TP53, NFKB1, MAPK3
hsa04152	AMPK signaling pathway	10.68	7.83E-06	11	PRKAA1, CCND1, FASN, AKT1, PPARG, CD36, PIK3CB, SIRT1, PRKAB1, FX1, MTR
hsa04141	Protein processing in endoplasmic reticulum	10.68	1.22E-04	11	MAPK10, MAPK9, XBP1, MAPK8, HSPA5, DDIT3, BCL2, EIF2AK3, BAX, EIF2S1, NFE2L2
hsa04014	Ras signaling pathway	10.68	0.0012353	11	MAPK10, MAPK9, MAPK8, HGF, MAPK1, AKT1, PIK3CB, MET, NFKB1, MAPK3, VEGFA
hsa04630	Jak-STAT signaling pathway	9.71	1.89E-04	10	IL10, IL4, IL6, CCND1, MYC, STAT3, AKT1, STAT6, PIK3CB, IL2
hsa04664	Fc epsilon RI signaling pathway	8.74	4.31E-06	9	MAPK10, IL4, MAPK9, MAPK8, MAPK1, AKT1, PIK3CB, TNF, MAPK3

### 3.4. Compound target pathway network construction

**Figure 5.**
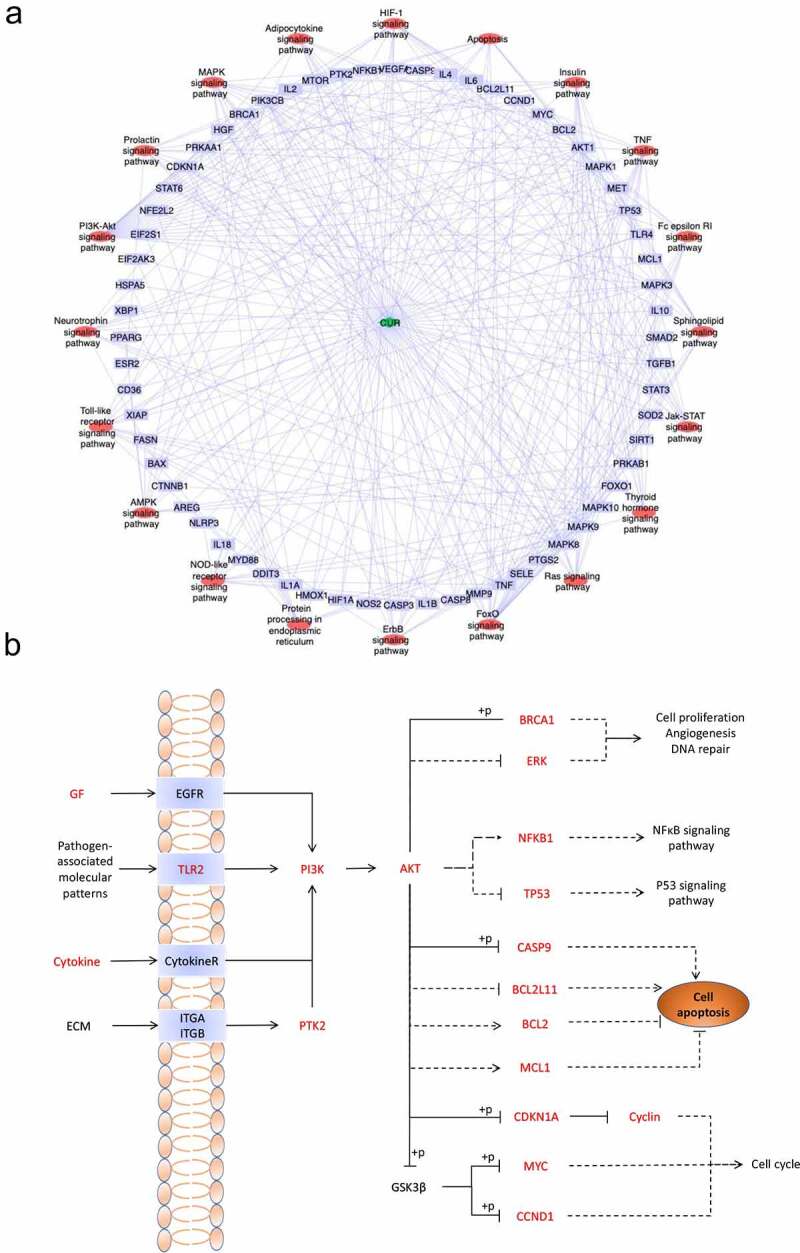
(a) KEGG pathway-target network diagram of CUR against CP-AKI. Red ellipse nodes represent enriched KEGG pathways, purple rectangle nodes indicate the targets, and the green diamond node indicates CUR. (b) Schematic drawing of the PI3K-Akt signaling pathway. The red font labels show the targets of CUR against CP-AKI involved in the PI3K-Akt signaling pathway

To clarify the relationships between the targets and pathways, the CUR, targets, and critical pathways were used to construct the compound target pathway (CTP) network (Figure 5(a)). As the most obviously enriched pathway by gene ratio (23.3%), the PI3K-Akt signaling pathway is primarily associated with cell proliferation and apoptosis (Figure 5(b)), and the identified targets shown in red font were associated with CUR against AKI.

### 3.5. CUR reduces CP-induced apoptosis in HK-2 cells

**Figure 6.**
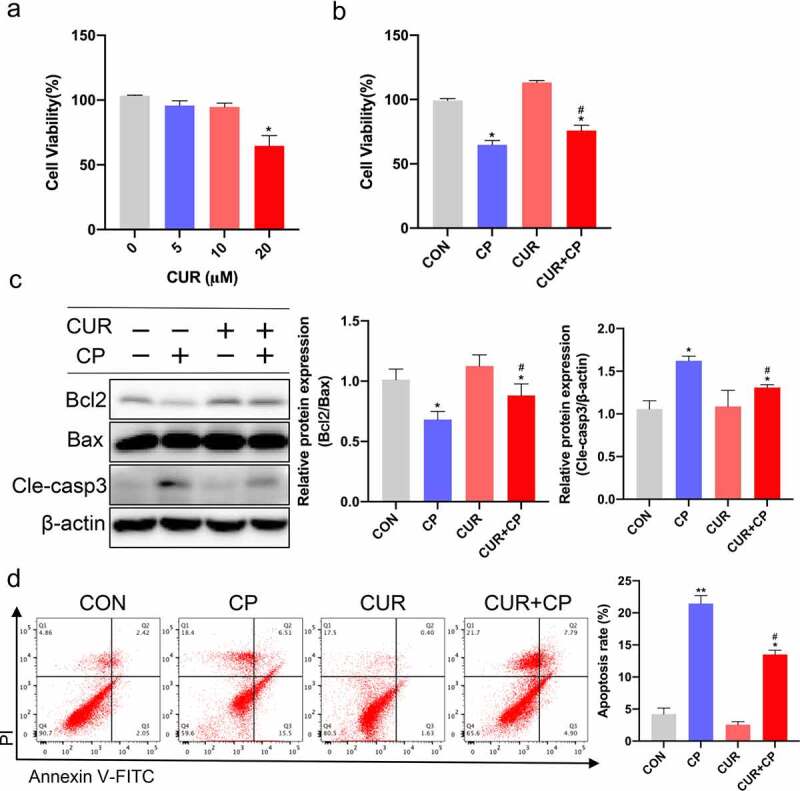
CUR inhibited CP-induced HK-2 cells apoptosis. (a) The cell viability of HK-2 cells exposed to different concentrations of CUR for 24 h was detected using CCK-8 analysis. Data are means ± SD (n = 7). * vs 0 μM: *p < 0.05. (b) Cell viability was evaluated by CCK-8 in HK-2 cells stimulated with CUR and CP for 24 h. Data are means ± SD (n = 7). *vs CON group: *p < 0.05; ^#^vs CP group: ^#^*p* < 0.05. (c) Protein levels of Bcl2, Bax, and cleaved caspase-3 (Cle-casp3) were measured by Western blots in HK-2 cells treated with CP or CUR for 24 h, and quantitatively analyzed. (d) Cell apoptosis was detected by flow cytometry in HK-2 cells after being treated with CP and CUR. Data are means ± SD (n = 3). HK-2 cells treated with 0.1% DMSO solvent served as CON group. *vs CON group: *p < 0.05, **p < 0.01; ^#^vs CP group: ^#^*p *< 0.05

To determine the appropriate concentration of CUR applied to renal tubular epithelial cells (TECs), human kidney tubular cells (HK-2) were treated with various concentrations of CUR for 24 h. The CCK-8 assay showed that CUR concentrations up to 20 μM caused a significant decrease in cell viability, and CUR concentrations of 0–10 μM had no cytotoxic effect on HK-2 cells (Figure 6(a)). Thus, for subsequent experiments, we selected 10 μM CUR to administer for 24 h. To investigate the effect of CUR on CP-AKI *in vitro*, HK-2 cells were treated with 20 µM CP for 24 h to establish the CP-AKI cell model [33–35]. In addition, these cells were administered 10 μM CUR for 24 h. The CCK-8 assay showed that CUR treatment partially reversed the decrease in cell viability caused by CP (Figure 6(b)). Notably, apoptosis is associated with the pathogenesis of CP-AKI [6]. In this context, enrichment analysis of the GO bioprocess and KEGG indicated that cell apoptosis is a crucial mechanism of CP-AKI development. Thus, we investigated the anti-apoptotic effect of CUR in HK-2 cells. HK-2 cells were treated with 20 μM CP and 10 μM CUR for 24 h. Then, WB was used to detect the ratio of the anti-apoptotic protein, Bcl2, and the pro-apoptotic protein, Bax. A decrease in the Bcl2/Bax ratio is a well-known marker of apoptosis [41]. CASP3 was the core target of CUR against CP-AKI, and the activation of CASP3 (cleaved caspase-3) indicates the occurrence of apoptosis. We found that CUR reversed the decrease in Bcl2/Bax ratio and increase in caspase-3 levels induced by CP treatment (Figure 6(c)). Flow cytometry analysis further confirmed that CUR inhibited apoptosis caused by CP *in vitro* (Figure 6(d)). These results indicate that CUR ameliorates apoptosis induced by CP in HK-2 cells.

### 3.6. CUR exerts anti-apoptosis effects via the Akt signaling pathway in HK-2 cells stimulated with CP

**Figure 7.**
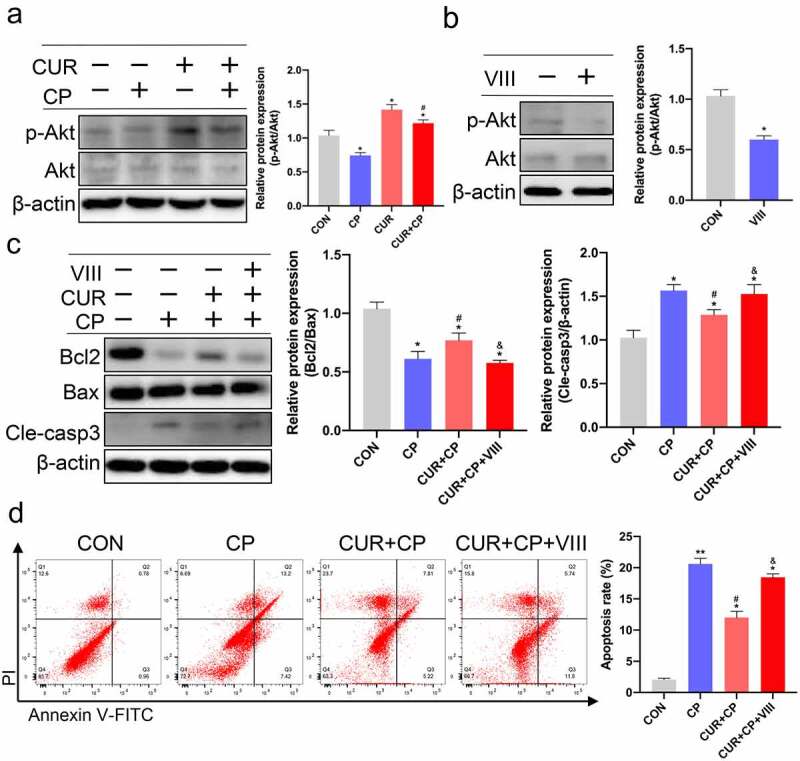
CUR exerts anti-apoptosis effects via the Akt signaling pathway in HK-2 cells. (a) The protein levels of phosphorylated Akt (p-Akt, Ser473) and total Akt (Akt)were measured by Western blotting in HK-2 cells treated with CP and CUR, and quantitatively analyzed. (b) Western blots detected p-Akt and Akt in HK-2 cells stimulated with VIII, and quantitatively analyzed. (c) Western blots evaluated protein expression of Bcl2, Bax, and cleaved caspase-3 in HK-2 cells stimulated with CP, CUR, and VIII, then quantitatively analyzed. (d) Apoptosis was analyzed by flow cytometry in HK-2 cells incubated with CP, CUR, and VIII. Data are means ± SD (n = 3). HK-2 cells treated with 0.1% DMSO solvent served as CON. *vs CON group: *p < 0.05, **p < 0.01; ^#^vs CP group: ^#^*p* < 0.05; ^&^vs CUR+CP group: ^&^*p* < 0.05

The KEGG enrichment analysis results revealed that the PI3K-Akt signaling pathway was the most significantly enriched and included 24 target proteins (Figures 4**(b), 4(c))**. The molecular docking results also showed that CUR had the best binding affinity to AKT1. AKT1, the major Akt isoform, is a hub member of the PI3K-Akt signaling pathway [42]. Therefore, we targeted Akt signaling to explore the anti-apoptotic effect of CUR on HK-2 cells treated with CP. First, we evaluated the protein expression of p-Akt and Akt in HK-2 cells treated with CP or CUR. WB indicated that CP markedly reduced Akt phosphorylation compared to that observed in the CON group, while CUR could obviously rescue the decreased levels of Akt phosphorylation induced by CP (Figure 7(a)). This result indicates that CUR could activate Akt signaling in HK-2 cells. Further, we explored whether the anti-apoptotic effect of CUR on CP-AKI was mediated by Akt signaling. Briefly, HK-2 cells were treated with a specific inhibitor of Akt activity, VIII. WB showed that VIII successfully inhibited Akt phosphorylation (Figure 7(b)). Subsequently, HK-2 cells were stimulated with CP, CUR, and VIII. WB indicated that the decreased Bcl2/Bax ratio and increased cleaved caspase-3 levels induced by CP could be reversed by CUR. In parallel with the inactivation of Akt, VIII clearly abolished the rescued effects of CUR on Bcl2/Bax ratio and cleaved caspase-3 expressions (Figure 7(c)). Likewise, flow cytometry analysis confirmed that VIII markedly aggravated the apoptotic rate of HK-2 cells treated with CUR and CP (Figure 7(d)). These results indicate that CUR inhibits CP-induced HK-2 cell apoptosis by activating Akt signaling.

## 4. Discussion

In this study, the crucial mechanism of CUR against CP-AKI was investigated by combining a network pharmacology approach and experimental validation. We found 106 potential targets of CUR against CP-AKI, and among these targets, ten targets were identified as the core targets by PPI network analysis. Furthermore, CUR showed good binding to the following targets with the highest degree value: AKT1, TP53, CASP3, and HIF1A, among which AKT1 showed the best binding affinity. The common targets were mainly involved in cellular response to chemical stress, cellular response to oxidative stress, and regulation of apoptotic signaling pathway. In addition, the PI3K-Akt signaling pathway was the most significant pathway in which shared targets were enriched. Our experiments further verified that CUR exerts an anti-apoptotic effect through activating the Akt signaling pathway in CP-AKI *in vitro*.

CUR has been widely used as an intervention for kidney diseases in animal and cell models over the past decade [43–46]. As a phenolic compound, CUR adheres to the five rules of Lipinski’s, demonstrating that CUR has good pharmacokinetic properties [39]. We collected 176 potential CUR targets from the HERB database, which integrates multiple TCM databases and supports TCM data based on a high-throughput experiment and reference-guidance, guaranteeing the credibility of targets related to CUR. By analyzing the degree value of the PPI network, ten core targets were acquired, including AKT1, TP53, CASP3, HIF1A, IL6, TNF, MYC, STAT3, VEGFA, and MAPK3. These targets had a higher degree value, indicating that they might play a crucial role in the entire interaction network of targets of CUR against CP-AKI.

According to molecular docking, among the four core targets with high degree value, CUR and AKT1 exhibited the lowest binding energy of – 9.5 kcal/mol, indicating the best binding activity, which aligned with the highest degree value of AKT (93). There are three Akt isoforms (AKT1–3), with AKT1 being the essential subtype. AKT1 is a hub member of the PI3K-Akt signaling pathway [47]. CUR exerts a renoprotective effect by ameliorating apoptosis in rats with rhabdomyolysis by activating Akt signaling [48]. Besides AKT1, the binding affinity of CUR with HIF1A also showed a strong binding energy (–8.9 kcal/mol). Hypoxia-inducible factor-1a (HIF1A) is a transcription factor that modulates cellular responses to tissue hypoxia [49]. HIF1A plays a vital role in kidney diseases induced by ischemia and nephrotoxicity [50]. *Panax notoginseng* saponins can activate HIF1A signaling to inhibit the mitochondrial apoptosis pathway, thereby reducing CP-induced acute renal injury [51]. In addition, TP53 (–6.6 kcal/mol) presented a good binding affinity with CUR. TP53 was upregulated and phosphorylated early during CP treatment, and the inhibition of TP53 dramatically attenuated CP-induced apoptosis in cultured rat TECs [52]. CASP3 (caspase 3) is a major executioner protein of proteolytic degradation during apoptosis. Previous study demonstrated that CP-induced renal cell apoptosis is partially dependent on CASP3 [53]. Consistent with previous studies, our data of molecular docking showed that CUR could bind good to CASP3 (–6.7 kcal/mol) and reversed the increased levels of cleaved caspase-3 induced by CP treatment in HK-2 cells. These results revealed that the drug molecule might directly bind to these core targets with a high and stable affinity and provided evidence for the crucial role of these core targets in CP-AKI therapy. The docking results verified the accuracy of the PPI network analysis results for identifying the core targets. More attention should thus be paid to these core targets in the treatment of CP-AKI in future research.

CUR has been reported to significantly alleviate CP-induced nephrotoxicity in rats at the ultrastructural and molecular levels. CUR reduced tubular necrosis and inflammation in the peritubular area, while in tubular epithelial cells, CUR prevented oxidative stress and apoptosis [54,55]. Consistent with those studies, our GO enrichment analysis showed that the primary biological processes of CUR against CP-AKI included cellular response to oxidative stress and the apoptotic signaling pathway. In addition, KEGG pathway enrichment analysis suggested that the PI3K-Akt signaling pathway was the most significantly enriched. Importantly, this study verified that CUR could inhibit apoptosis in CP-treated HK-2 cells by activating Akt signaling. The pharmacological inhibition of Akt significantly abolished the anti-apoptotic effect of CUR on CP-AKI *in vitro*. According to previous reports, the PI3K-Akt signaling pathway plays a significant role in promoting cell survival by inhibiting the intrinsic apoptotic pathway [56,57]. Akt signaling activation could exert a protective effect against high glucose-induced HK-2 cell injury [58]. Dexmedetomidine activated the PI3K-Akt signaling pathway to alleviate endoplasmic reticulum stress-induced apoptosis, thereby attenuating CP-AKI [59]. Undoubtedly, our network pharmacology mining results are consistent with those of previous studies, and our data from *in vitro* experiments coincide with the literature.

Although many studies have reported the possible mechanisms and intervention measures in CP-AKI, our findings are still important: 1. Based on the exploration of the pharmacology network, we associated the PI3K-Akt signaling pathway with CUR treatment in CP-AKI. Further, we successfully demonstrated that CUR may be used as an Akt activator to treat CP-AKI *in vitro* and verified the accuracy of network pharmacology analysis. 2. Distinct from other CP-AKI-related studies focused on the validations of some exact signaling pathways [55,60]. Our study is based on a network pharmacology approach that can identify multiple promising signaling pathways, such as FOXO, TNF, MAPK and HIF-1 signaling pathways. These pathways also deserve further exploration as therapeutic options for CP-AKI. 3. In other kidney diseases, CUR may regulate these pathways to exert protective effects.

Altogether, these results demonstrate that CUR may play a renoprotective role by inhibiting TEC apoptosis via the activation of the Akt signaling pathway in CP-AKI. This study had some limitations as multiple complicated pathological pathways are involved in the mechanism of CUR against CP-AKI. In addition, apart from the severe damage to the proximal tubules, cisplatin can cause an injury in the distal tubules, which is also involved in the development of AKI [61]. Further investigations are required, including explorations on other mechanisms and experiments in the distal tubules as well as in animal models.

## 5. Conclusion

In conclusion, we identified the core targets and critical pathways of CUR against CP-AKI using a pharmacological approach. Further, we confirmed that CUR inhibits cell apoptosis by activating Akt signaling in CP-AKI cell models. These results suggest that CUR is a promising and effective multitarget medication for CP-AKI.

## Figures and Tables

**Table:** 

CP	Cisplatin
CUR	Curcumin
AKI	Acute kidney injury
PPI	Protein-protein interaction
GO	Gene Ontology
KEGG	Kyoto Encyclopedia of Genes and Genomes
OMIM	Online Mendelian Inheritance in Man
Hacc	Hydrogen bond acceptors
Hdon	Hydrogen bond donors
BP	Biological process
CC	Cell composition
MF	Molecular function
TECs	Renal tubular epithelial cells
HK-2	Human kidney tubular epithelial cell
CCK-8	cell counting kit‑8
WB	Western blots
CON	Control
Cle-casp3	Cleaved caspase-3

## Data Availability

The data used to support the findings of this study are included within the article.

## Highlights

- Network pharmacology and experimental validation were used to explore the mechanism of CUR against CP-AKI.
- Among the core targets of CUR against CP-AKI, AKT1 binds the best to CUR.
- PI3K-Akt signaling pathway is significantly enriched based on KEGG pathway enrichment analysis.
- CUR inhibits apoptosis by activating the Akt signaling pathway in CP-AKI *in vitro*.
